# Radiographic study of iliac screw passages

**DOI:** 10.1186/1749-799X-9-40

**Published:** 2014-05-23

**Authors:** Bin Liu, Jiwei Wang, Liyan Zhang, Wei Gan

**Affiliations:** 1 Department of Orthopaedics, Sixth Affiliated Hospital of Wenzhou Medical University, Lishui 323000, Zhejiang Province, China

**Keywords:** Ilium, Photogrammetry, Clinical research

## Abstract

**Background:**

The optimal iliac screw path was determined to provide references for lumbosacral-pelvic reconstruction.

**Methods:**

Radiographic data of 100 patients with normal pelvis were selected for this study. Four paths were designed. Paths A, B, and C were from the starting point of the crossing point of the chiotic line and posterior iliac crest (CLIC, located at 24.0 mm above the posterior superior iliac spine) to the upper edge of the acetabulum, anterior inferior iliac spine, and acetabulum center, respectively. Path D was from the starting point of the posterior superior iliac spine to the anterior inferior iliac spine. The lengths of the different paths of screw passage and bone plate thicknesses of two narrow places were measured and analyzed.

**Results:**

Paths A, B, and D were approximately equal in length, but the thickness of the iliac plate in path A was significantly thicker than those in paths B and D. No significant difference was found between the iliac thickness of paths A and C, but the passage length of path A was significantly longer than that of path C.

**Conclusion:**

Path A had the longest passage length and thickest iliac plate and could accommodate the relatively longest and thickest iliac screw. Thus, path A was the optimal iliac screw passage.

## Introduction

Iliac screw fixation is used in the reconstruction of spinal-pelvic stability [1-6]. Iliac screws are important in achieving strong fixation, maintenance of orthopedic position, and protection of sacral screw [7]. The lumbosacral stabilization and fixation of iliac screw is satisfactory [3,8-10]. In recent years, the implementation of lumbosacral-pelvic reconstruction using iliac screw has become increasingly accepted by spine surgeons. Many different paths of iliac screw passage are available, but no relatively consistent scientific path exists. Thus, establishing an effective and accepted iliac screw passage for the development of lumbosacral-pelvic reconstruction is crucial. Computed tomography (CT) and radiographic analysis were employed to determine the optimal iliac screw path, thereby providing references for lumbosacral-pelvic reconstruction.

## Materials and methods

### Case selection

One hundred outpatients or inpatients underwent pelvic CT (GE Brightspeed16 slice spiral CT, Milwaukee, WI, USA) scan and three-dimensional (3D) reconstruction without positive performance for various reasons from February 2009 to June 2012. A total of 58 males and 42 females, aged 19 to 65 years (average age 42.2 years), were involved in this study. Their radiographic data were evaluated. The inclusion criteria were adults with pelvis under normal development and absence of abnormalities, such as iliac bone deformities, tumors, and trauma fractures. This study was conducted in accordance with the declaration of Helsinki. This study was conducted with approval from the Ethics Committee of the Sixth Affiliated Hospital of Wenzhou Medical College. Written informed consent was obtained from all participants.

### Design method

A line was drawn from the upper edge of the eminentia iliopubica to the closest point of the front edge of the auricular surface to the posterior iliac crest. This line, which was called the chiotic line, divided the posterior iliac crest into the front and back sections. The crossing point of the chiotic line and posterior iliac crest was called the CLIC point, which was located 24.0 mm above the posterior superior iliac spine (PSIS).

**Figure 1:**
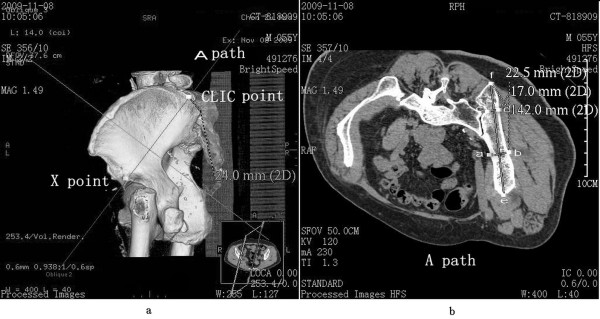
**Path A (CLIC point to the upper edge of the acetabulum) diagram. (a)** Direction diagram of path A, with *X* as the upper edge of the acetabulum; **(b)** diagram of length of path and thickness of the ilium, with the distance between *e* and *f* as the total length of path, the distance between *a* and *b* as the thickness of the first narrow place, and the distance between *c* and *d* as the thickness of the second narrow place.

**Figure 2:**
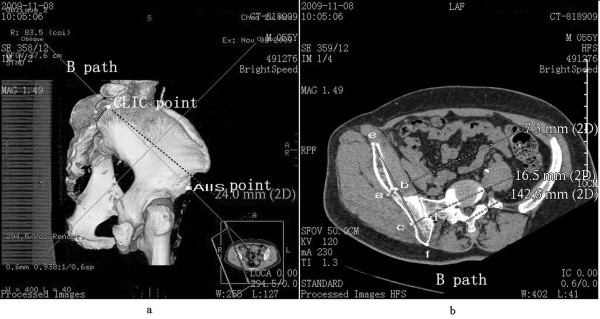
**Path B (CLIC point to the AIIS) diagram. (a)** Direction diagram of path B; **(b)** diagram of length of path and thickness of the ilium, with the distance between *e* and *f* as the total length of path, the distance between *a* and *b* as the thickness of the first narrow place, and the distance between *c* and *d* as the thickness of the second narrow place.

**Figure 3:**
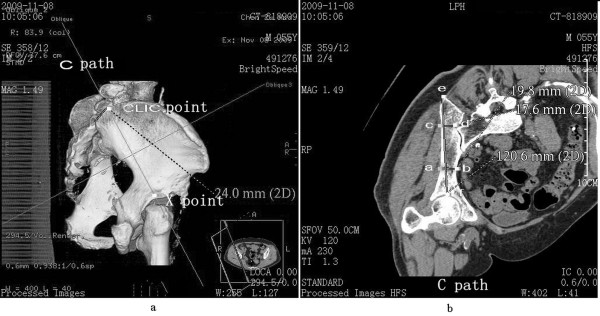
**Path C (CLIC point to the acetabular center) diagram. (a)** Direction diagram of path C, with *X* as the acetabular center; **(b)** diagram of length of path and thickness of the ilium at the location of the narrow places, with the distance between *e* and *f* as the total length of path, the distance between *a* and *b* as the thickness of the first narrow place, and the distance between *c* and *d* as the thickness of the second narrow place.

**Figure 4:**
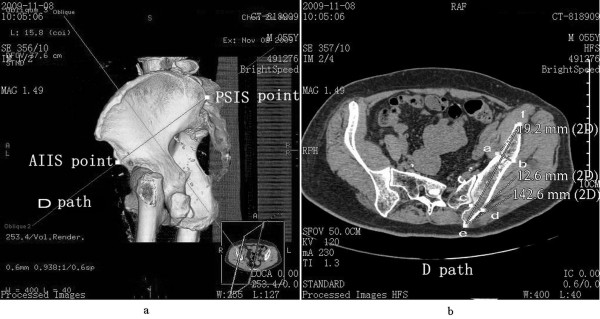
**Path D (PSIS to AIIS) diagram. (a)** Direction diagram of path D; **(b)** diagram of length of path and thickness of the ilium at the location of the narrow places, with the distance between *e* and *f* as the total length of path, the distance between *a* and *b* as the thickness of the first narrow place, and the distance between *c* and *d* as the thickness of the second narrow place.

Four iliac screw paths were designed. Paths A, B, and C were from the starting point of the CLIC to the upper edge of the acetabulum, anterior inferior iliac spine (AIIS), and acetabulum center, respectively. Path D was from the starting point of the PSIS to the AIIS. Specific methods were as follows:

1. Path A represented the connection and screw passage direction from the CLIC point to the upper edge of the acetabulum (Figure 1a). A 3D reconstruction image was truncated along the direction of path A to obtain the sectional view. The total bone length of path A was measured as the length of the passage. Two narrow places were found in the connection line. The vertical line of path A was constructed in the first narrow place, which was close to the CLIC point. The total thickness of the iliac cortical bone and cancellous bone was measured as the thickness of the first narrow place. The vertical line of path A was constructed in the second narrow place, which was close to the upper edge of the acetabulum. The total thickness of the iliac cortical bone and cancellous bone was measured as the thickness of the second narrow place (Figure 1b).

2. Path B represented the connection and screw passage direction from the CLIC point to the AIIS (Figure 2a). A 3D reconstruction image was truncated along the direction of path B to obtain the sectional view. The total bone length of path B was measured as the length of passage. Two narrow places were found in the connection line. The vertical line of path B was constructed in the first narrow place, which was close to the CLIC point. The total thickness of the iliac cortical bone and cancellous bone was measured as the thickness of the first narrow place. The vertical line of path B was constructed in the second narrow place, which was close to the AIIS. The total thickness of the iliac cortical bone and cancellous bone was measured as the thickness of the second narrow place (Figure 2b).

3. Path C represented the connection and screw passage direction from the CLIC point to the acetabular center (Figure 3a). A 3D reconstruction image was truncated along the direction of path C to obtain the sectional view. The total bone length of path C was measured as the length of passage. Two narrow places were found in the connection line. The vertical line of path C was constructed in the first narrow place, which was close to the CLIC point. The total thickness of the iliac cortical bone and cancellous bone was measured as the thickness of the first narrow place. The vertical line of path C was constructed in the second narrow place, which was close to the acetabular center. The total thickness of the iliac cortical bone and cancellous bone was measured as the thickness of the second narrow place (Figure 3b).

4. Path D represented the connection and screw passage direction of the PSIS and AIIS (Figure 4a). A 3D reconstruction image was truncated along the direction of path D to obtain the sectional view. The total bone length of path D was measured as the length of passage. Two narrow places were found in the connection line. The vertical line of path D was constructed in the first narrow place, which was close to the PSIS. The total thickness of the iliac cortical bone and cancellous bone was measured as the thickness of the first narrow place. The vertical line of path D was constructed in the second narrow place, which was close to the AIIS. The total thickness of the iliac cortical bone and cancellous bone was measured as the thickness of the second narrow place (Figure 4b).

### Observation indexes and methods

Patients underwent pelvic CT scan in a GE Brightspeed16 slice spiral CT system (USA) with spacing of 1.25 mm and 3D computer reconstruction. The distance between the two points was directly measured, with an accuracy of 0.1 mm. The lengths of screw passage and thicknesses of bone plates in two narrow places in different paths were measured and analyzed. Each radiographic datum was repeatedly measured twice within 1 week by the same surveyor, and the mean value was used as the final result.

### Statistical analysis

SPSS13.0 software was used in this study. The measured data in accordance with normal distribution were presented as $\overset{¯}{x}\pm s$. Various paths were compared using independent-groups *t* test, with an inspection level of bilateral alpha = 0.05. *P* < 0.05 was considered statistically significant.

## Results

Four paths with different entrance points and screw passages were measured and analyzed. Paths A (from the CLIC point to the upper edge of the acetabulum), B (from the CLIC point to the AIIS), and D (from the PSIS to the AIIS) had similar lengths of screw passage, but the thickness of iliac plate in path A was significantly thicker than those in paths B and D. No significant difference was found between the iliac thickness of paths A and C (from the CLIC point to the acetabular center), but the passage length of path A was significantly longer than that of path C. Among the paths from the CLIC, path A had the longest passage length and thickest iliac plate, and could accommodate the relatively longest and thickest iliac screw. Thus, path A was significantly superior to the other three paths. The lengths of passages and thicknesses of bone plates in two narrow places in the different paths are shown in Table 1.

**Table 1:** Lengths of four paths of screw passage and bone plate thicknesses of two narrow places

**Path**	**Total length of screw passage (**$\overset{¯}{x}$ ***± s*, mm, *n*** **= 100)**	**Thickness of the first narrow place (**$\overset{¯}{x}$ ***± s*, mm, *n*** **= 100)**	**Thickness of the second narrow place (**$\overset{¯}{x}$ ***± s*, mm, *n*** **= 100)**
A	140.6 ± 5.9	17.3 ± 3.1	23.6 ± 2.6
B	138.1 ± 6.4*	14.3 ± 2.4**	12.8 ± 3.5***
C	118.9 ± 7.3^▲^	17.2 ± 2.7^▲▲^	22.8 ± 4.6^▲▲▲^
D	139.1 ± 5.6^△^	15.7 ± 2.8^△△^	20.5 ± 2.8^△△△^

Compared with path A, *t = 2.87, *P* < 0.01; **t = 7.65, *P* < 0.001; *** *t* = 24.77, *P* < 0.001; ^▲^*t* = 23.12, *P* < 0.001; ^▲▲^*t* = 0.24, *P* > 0.05; ^▲▲▲^*t* = 1.51, *P* > 0.05; ^△^*t* = 1.84, *P* > 0.05; ^△△^*t* = 3.83, *P* < 0.001; ^△△△^*t* = 8.11, *P* < 0.001.

## Discussion

Lumbosacral-pelvic fixation without pelvic intensive long segment fusion had a clinically higher non-fusion rate, regardless of the number of sacral fixation screws. Application of iliac screw in combination with lumbar pedicle screw and rod could produce a better effect in maintaining lumbosacral-pelvic stability. McCord et al. [8] detected 10 different lumbosacral fixation systems, and confirmed that the iliac rods or screws have the strongest capacity selection of high load.

In the clinical application of iliac screws, orthopedic surgeons have difficulties in avoiding intraoperative nerve injury, blood vessels, and other important structures. Orthopedic surgeons also experience difficulties in the safe and effective implantation of iliac screws. As of this writing, the entrance point and path of iliac screws have no uniform standard. Two sclerotin-rich columnar zones are found in the ilium [11-13], which can provide anchors for iliac screws. Two high-density bone areas, which are located in the sacroiliac joint and sciatic notch, also exist in the lower columnar iliac screw path. Biomechanics research showed that the iliac screw can obtain a reliable anchor once across the second high-density bone area [14]. Similar to this study, two narrow places in iliac screw paths also exhibited anchoring effects.

The entrance point of the iliac screw is controversial. Berry et al. [15] believed that the optimal entrance point is the PSIS, whereas Schwend et al. [16] believed that the screws should enter below the PSIS. By contrast, Schildhauer et al. [11] believed that entry above the PSIS is more ideal. The entrance point of the iliac screw in the CLIC point can effectively avoid wound infection and implant exposure caused by local skin compression and necrosis, which are due to screw-tail protrusion when the PSIS is used as the entrance point. Moreover, sclerotin removal of the PSIS is unnecessary. This study showed that the iliac screw passage from the CLIC point to the upper edge of the acetabulum was the longest and thickest, and could withstand the largest tensile force. Thus, this entry point was the optimal iliac screw passage. However, the effects of several factors, such as gender and regional differences, should be investigated. Preoperative screwing guidance to CT 3D reconstruction is suggested to enhance immobility and effectiveness.

## Conclusions

Iliac screw path has an important value for lumbosacral-pelvic reconstruction. Of all paths in our design, path A (from CLIC point to the upper edge of the acetabulum) had the longest passage length and thickest iliac plate and could accommodate the relatively longest and thickest iliac screw. Thus, path A was the optimal iliac screw passage. In the future, we also need to be confirmed in the clinical work.

## Competing interests

The authors declare that they have no competing interests.

## Authors' contributions

BL reviewed the database and prepared the manuscript. JW and WG carried out the statistical analysis and assisted with preparation of the manuscript. LZ assembled the database and supervised the writing of the whole paper. All authors read and approved the final manuscript.
